# Human PSC-Derived Hepatocytes Express Low Levels of Viral Pathogen Recognition Receptors, but Are Capable of Mounting an Effective Innate Immune Response

**DOI:** 10.3390/ijms21113831

**Published:** 2020-05-28

**Authors:** Lena Fischer, Baltasar Lucendo-Villarin, David C. Hay, Cliona O’Farrelly

**Affiliations:** 1 School of Biochemistry and Immunology, Trinity Biomedical Sciences Institute, Trinity College Dublin, Dublin 2, Ireland; lfische3@exseed.ed.ac.uk; 2 Centre for Regenerative Medicine, University of Edinburgh, Edinburgh EH16 4UU, UK; blucendo@exseed.ed.ac.uk; 3 School of Medicine, Trinity Biomedical Science Institute, Trinity College Dublin, Dublin 2, Ireland

**Keywords:** stem cells, hepatocyte-like cells, innate anti-viral immunity, pattern recognition receptors (PRRs), metabolic switch

## Abstract

Hepatocytes are key players in the innate immune response to liver pathogens but are challenging to study because of inaccessibility and a short half-life. Recent advances in in vitro differentiation of hepatocyte-like cells (HLCs) facilitated studies of hepatocyte–pathogen interactions. Here, we aimed to define the anti-viral innate immune potential of human HLCs with a focus on pattern recognition receptor (PRR)-expression and the presence of a metabolic switch. We analysed cytoplasmic PRR and endosomal toll-like receptor (TLR)-expression, as well as activity and adaptation of HLCs to an inflammatory environment. We found that transcript levels of retinoic acid inducible gene I (RIG-I), melanoma differentiation antigen 5 (MDA5), and TLR3 became downregulated during differentiation, indicating the acquisition of a more tolerogenic phenotype, as expected in healthy hepatocytes. HLCs responded to activation of RIG-I by producing interferons (IFNs) and IFN-stimulated genes. Despite low-level levels of TLR3, receptor expression was upregulated in an inflammatory environment. TLR3 signalling induced expression of proinflammatory cytokines at the gene level, indicating that several PRRs need to interact for successful innate immune activation. The inflammatory responsiveness of HLCs was accompanied by the downregulation of cytochrome P450 3A and 1A2 activity and decreased serum protein production, showing that the metabolic switch seen in primary hepatocytes during anti-viral responses is also present in HLCs.

## 1. Introduction

The liver has long been perceived exclusively as a metabolic organ where hepatocytes, the main cell type of the liver, are primarily responsible for nutrient metabolism, drug detoxification, and serum protein synthesis. This view is being enriched as immunological functions for the liver are being discovered, in particular for hepatocytes. Hepatocytes are now known to be important detectors of infection and inflammation, as well as major producers of acute phase proteins, complement components, and antimicrobial peptides. They thus play critical roles in mediating systemic and local inflammatory and innate immune functions [1,2]. However, this antimicrobial and antiviral potential has to be tempered by the requirement for tolerance to harmless microbial products from gut commensal organisms. Research in this field is slow owing to inaccessibility of human liver tissue. Advances in in vitro differentiation of pluripotent stem cells (PSCs) into hepatocytes are transforming research in this area [3,4,5,6,7,8]. These models have already permitted more detailed studies of host–pathogen interactions, leading to new insights into the importance of hepatocytes in innate immune responses against viruses such as hepatitis C (HCV) and hepatitis B (HBV) [9,10,11,12]. These early studies also showed advantages of induced pluripotent stem cells (iPSCs) and human embryonic stem cells (hESCs) over hepatoma cell lines, which had classically been used for host–pathogen studies and which lack toll-like receptor 3 (TLR3) and show varying and often diminished retinoic acid inducible gene I (RIG-I) and type III interferon (IFN) responses [9,13,14,15]. As such, the widely used hepatoma cell line Huh7 is defective for TLR3 signalling owing to insufficient expression of TLR3 [14]. Huh7.5 cells, which are derived from Huh7 cells, show further defects with an additional disabling mutation in the RIG-I gene and only low-level expression of melanoma differentiation antigen 5 (MDA5) [16]. While both Huh7 and Huh7.5 cells are important tools for studying the HCV-replication cycle in vitro, they are poor physiological models for the study of host defense mechanisms and the complex interplay between virus and host.

Innate defense mechanisms in hepatocytes include several viral detection molecules and downstream signalling pathways, which are either constitutively expressed or are upregulated in the event of viral infection. Initial viral detection by the cytoplasmic pattern recognition receptors (PRRs) RIG-I and MDA5 has been demonstrated in iPSC-derived hepatocyte like cells (HLCs), as well as downstream signalling and interferon (IFN) responses [9,17]. However, the role of TLRs is more difficult to assess. Primary hepatocytes are thought to constitutively express low levels of RNA for all TLRs, while the presence of TLR protein is limited [18,19,20]. The expression of individual TLR-proteins is tightly regulated in order to avoid excess immune responses in an organ that is constantly exposed to antigens of various origins such as food-derivatives and commensal organisms, but increased expression can be quickly triggered under inflammatory or infectious conditions [21].

Hepatocyte proliferation, survival, and cell death are regulated by tumor necrosis factor alpha (TNF-α); however, TNF-α also plays an important role in host defense. Hepatoma cell lines are characterised by deregulated proliferation and senescence and, therefore, provide limited suitability for studies on TNF-α signalling. Therefore, cells that are more primary in nature, such as HLCs, open up new possibilities to investigate the role of TNF-α in infection, proliferation, and carcinogenesis in human liver.

Metabolic changes that accompany innate immune mechanisms in the hepatocyte are likely to amplify the efficiency of pro-inflammatory and anti-viral activity [22]. In the healthy state, hepatocytes are responsible for drug detoxification, protein synthesis, and the metabolism of carbohydrates and lipids. Many metabolic functions are carried out by cytochrome P450 (CYP) enzymes that metabolise endo- and xenobiotic substrates. Some of the most abundant CYP-enzymes in the adult liver are CYP3A and CYP1A2. Adult hepatocytes also produce the serum protein albumin, a major component of blood plasma. When hepatocytes activate innate immune responses, these normal metabolic functions are downregulated [22,23,24]. The switch from a metabolic to defense mode allows the cell to dedicate all energy to fighting off infection.

Here, we sought to evaluate the innate anti-viral immune response of HLCs. We focused on receptors involved in the detection of RNA-viruses including cytoplasmic (RIG-I/MDA5) and endosomal receptors (TLR3 and 7) as well as IFN and TNF-α responses. After initial screening of PRR expression in two iPSC and hESC lines, we focused our work on the iPSC-line P106 based on the finding that TLR3 expression was higher in iPSCs compared with hESCs and, therefore, more closely resembles TLR3 mRNA levels in primary human hepatocytes (PHHs). We were particularly interested in whether stem cell-derived HLCs are able to upregulate TLRs in inflammatory conditions, as has been shown for PHHs. We analysed functional anti-viral innate immune signalling by release of CXC-motif chemokine 10 (CXCL10) upon stimulation with viral ligands. To determine whether TLR3-signalling is functional in HLCs, we blunted interfering pathways before analysing expression of CXCL10 and TNF-α. Finally, we studied if normal HLC metabolic function was altered under inflammatory conditions to determine the extent to which HLCs can raise an effective innate immune response. While the downregulation of CYP P450-activity and albumin production accompanies innate immune responses in PHHs [22,23,24], it is unknown whether stem cell-derived HLCs possess the same abilities. Previous studies on stem cell-derived HLCs reported reduced innate immune responses to viral infection, while the underlying mechanism for this has yet to be identified [25]. We demonstrate the upregulation of TLR receptors and a concomitant downregulation of normal metabolic functions under inflammatory conditions, demonstrating an effective metabolic switch and effective anti-viral response in stem cell-derived HLCs.

## 2. Results

### 2.1. Hepatocyte Differentiation of iPSCs

**Figure 1:**
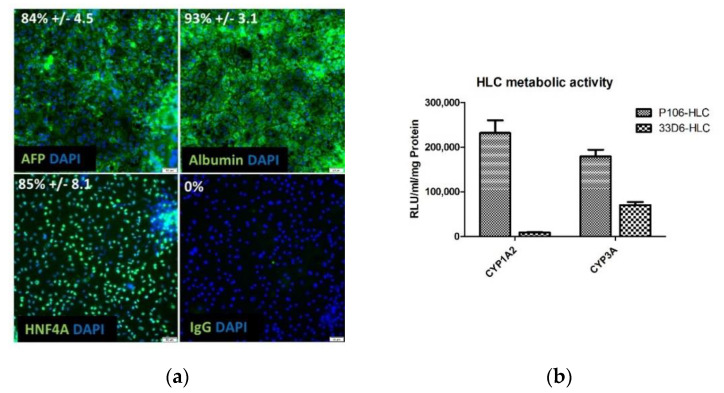
Characterisation of induced pluripotent stem cell (iPSC)-derived hepatocyte-like cells (HLCs) on d20 of differentiation. (**a**) Fixed cells (P106-HLCs) were stained for albumin, AFP, and HNF4α to assess hepatic characteristics and maturation state of HLCs. Immunofluorescence staining (40x magnification) shows that d20 HLCs co-express albumin and AFP representing mature and fetal characteristics (five fields of view per well with 400 cells, three wells per condition). (**b**) Metabolic activity. The metabolic activity of P106- and 33D6-HLCs was determined by measuring the activity of cytochrome P450 (CYP)1A2 and CYP3A in HLCs. CYP-activity was measured in RLU and normalised to ml and mg of total protein (*N* = 4). d20 HLCs are metabolically active, showing successful differentiation. P106-HLCs show improved metabolic activity compared with 33D6-HLCs. Results are shown as mean +/− SEM. AFP = alpha fetoprotein, DAPI = 4′,6-Diamidin-2-phenylindol, HNF4α = hepatocyte nuclear factor 4 alpha, RLU = relative light units.

iPSCs and hESCs were differentiated into HLCs using established procedures [26]. d20 HLCs morphologically resembled PHHs with a large cytoplasm to nucleus ratio and canaliculi formation (Figure S1a). Immunofluorescence staining of P106-HLCs showed that HLCs co-expressed mature albumin, 93% +/− 3.1 positive cells) and immature (AFP, 84% +/− 4.5 positive cells) hepatocyte markers confirming findings by others that, while stem-cell derived HLCs have attained a certain level of maturity, they retain some features of fetal hepatocytes (Figure 1a). HLCs were also metabolically active, showing Cyp1A2 and Cyp3A function (Figure 1b and Figure S1b). Comparison between the two iPSC lines showed that P106-derived HLCs had achieved improved metabolic function compared with 33D6-HLCs (Figure 1b and Figure S1b). In a previous study, Cameron et al. analysed CYP metabolic function of HLCs derived from different hESC lines and compared it to male and female PHHs [7]. CYP1A2 and CYP3A function closely resembled day 18 HLCs and male PHHs as previously described. [7].

### 2.2. Expression of Pattern Recognition Receptors (PRRs) across Different Stem Cell Lines

**Figure 2:**
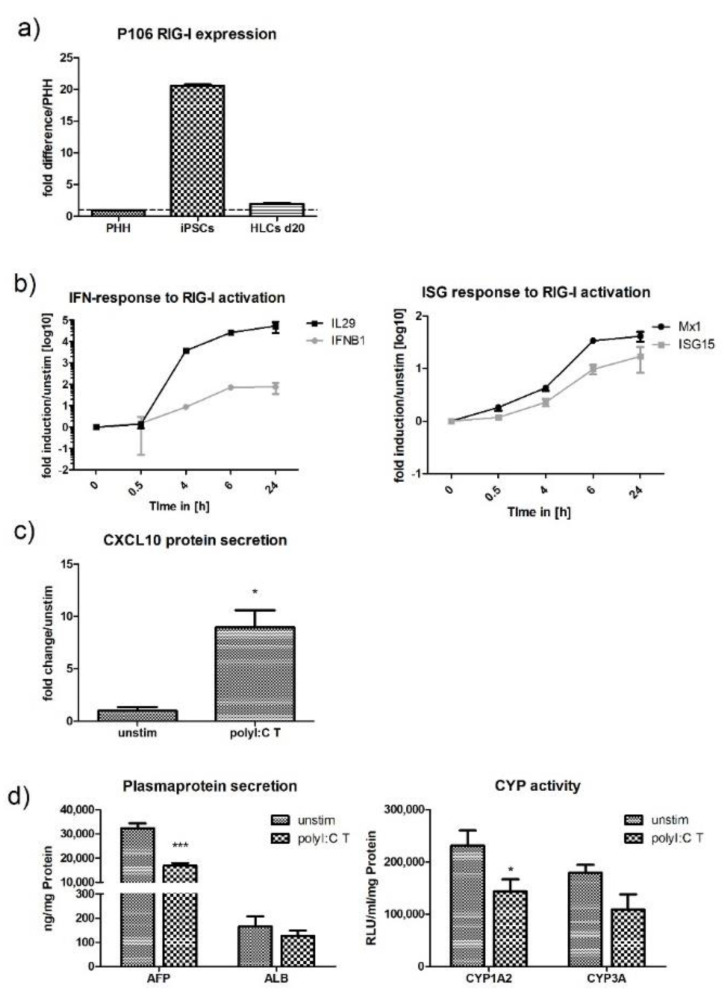
RIG-I expression and activation in P106-HLCs. (**a**) Comparison of baseline RIG-I mRNA expression in PHH, P106-iPSCs, and d20 P106-HLCs. Gene-expression was determined by qPCR, normalised to b2m, and compared to PRR gene-expression in primary human hepatocytes. iPSCs expressed high levels of RIG-I mRNA. Upon differentiation, RIG-I levels became downregulated and on d20 of differentiation were similar to PHH (*N* = 3). (**b**) P106-HLCs mount strong innate immune responses upon activation of RIG-I. qPCR data shows that RIG-I activation by polyI:C transfection induced type I and III IFN expression in a time-dependent manner, which then induced ISG-expression (ISG15, Mx1) (*N* = 3). (**c**) Expression of CXCL10 protein upon transfection with polyI:C. PolyI:C transfection induced significant expression of CXCL10 protein as determined by enzyme-linked immunosorbent assay (ELISA) 24 h after transfection (*N* = 3). (**d**) Effect of innate immune activation on hepatocyte metabolic activity. Cells were transfected with polyI:C for 16 h before CYP-activity and plasma protein production was measured and compared to unstimulated cells. The generation of an inflammatory environment induced downregulation of AFP and CYP1A2 when innate immune responses were activated (*N* = 3). Results are shown as mean +/− SEM. *p < 0.05 and ***p < 0.001. PHH = primary human hepatocytes, RIG-I = retinoic acid inducible gene I, HLCs = hepatocyte like cells, T = transfection, IFN = interferon, ISG = interferon stimulated gene, CXCL10 = C-X-C motif chemokine 10, AFP = alfa fetoprotein, ALB = albumin, CYP = cytochrome P450.

**Figure 3:**
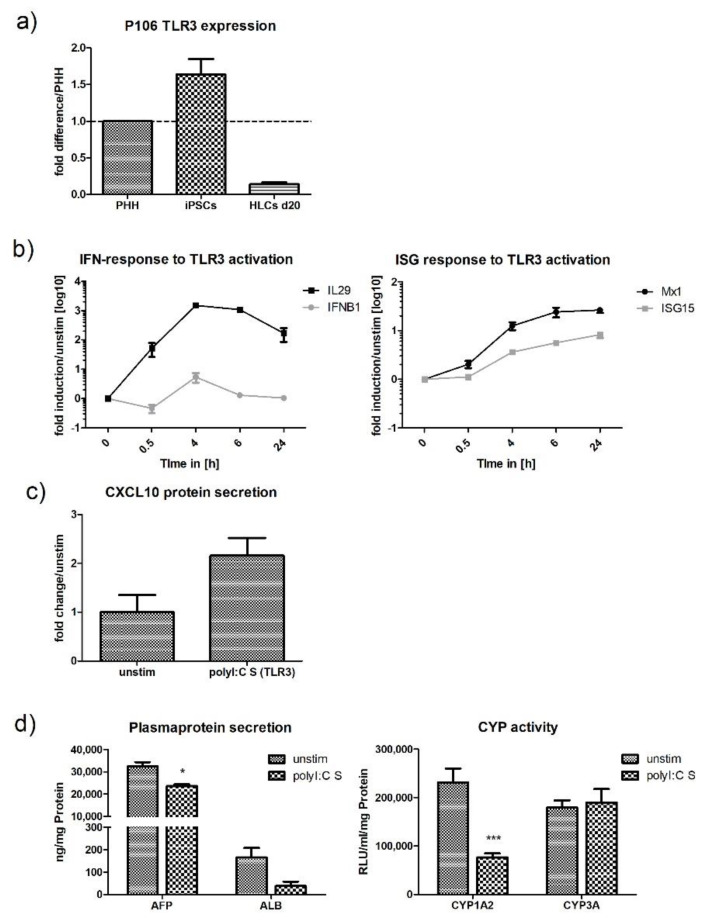
HLCs express low levels of TLR3 but can respond to viral ligand stimulation. (**a**) Comparison of baseline TLR3 mRNA expression in PHH, P106-iPSCs, and P106 HLCs on d20. Gene-expression was determined by qPCR, normalised to b2m, and compared to PRR gene-expression in PHHs. iPSCs expressed high levels of TLR3 mRNA, while levels in HLCs were lower compared with PHHs (*N* = 3). (**b**) P106-HLCs mount strong innate immune responses upon activation of TLR3 by polyI:C stimulation. PolyI:C stimulation induced type I and III IFN expression in a time-dependent manner, as determined by qPCR, and IFNs further induced ISG-expression (ISG15, Mx1) (*N* = 3). (**c**) Expression of CXCL10 protein upon stimulation with polyI:C. As determined by ELISA 24 h after stimulation, polyI:C induced the expression CXCL10 protein, although this was not significant (*N* = 3). (**d**) Effect of innate immune activation on hepatocytes metabolic activity. Cells were treated with polyI:C for 16 h before CYP-activity and plasma protein production was measured and compared to unstimulated cells. The generation of an inflammatory environment induced downregulation of AFP, ALB, and CYP1A2, while innate immune responses were activated (*N* = 3). Results are shown as mean +/− SEM. *p < 0.05, and ***p < 0.001. PHH = primary human hepatocytes, TLR3 = Toll-like receptor 3, HLCs = hepatocyte like cells. S = stimulation, IFN = interferon, ISG = Interferon stimulated genes, CXCL10 = C-X-C motif chemokine 10, AFP = alfa fetoprotein, ALB = albumin, CYP = cytochrome P450.

To compare the suitability of different stem cell lines for studies on anti-viral innate immunity, we first assessed gene expression of cytoplasmic and endosomal PRRs in different stem cell lines and their differentiated counterparts. The stem cell lines profiled were the two hESC-lines, Man12 (male) and H9 (female), and two iPSC-lines, P106 and 33D6 (male). Gene expression of the cytoplasmic receptors RIG-I and MDA5 as well as of the endosomal receptors TLR3, 7, and 9 was determined by qPCR (Figure 2a or Figure 3a and Figure S2). Comparison of PRR-expression between PSCs and HLCs was performed. High level expression of RIG-I, MDA5, and TLR3 was observed in the undifferentiated state, with continuous downregulation during differentiation. While cytoplasmic receptor expression was elevated compared with PHH, TLR3 mRNA was significantly downregulated in d20 HLCs. Notably, iPSC-derived HLCs of the P106 line expressed higher levels of TLR3 mRNA than hESC-derived HLCs or 33D6-HLCs, making these cells more suitable for subsequent experimentation (Figure 3a and Figure S2). TLR3 mRNA-expression in P106-HLCs was 4.3-fold higher than in H9-HLCs, 2.3-fold higher than in Man12-HLCs (statistical significance reached), and 1.3-fold higher than in 33D6-HLCs. In this study, we could not detect mRNA for TLR7 or 9 in PHHs, iPSCs, or HLCs.

P106-derived HLCs were used for all further experiments. This decision was based on two factors. Firstly, although TLR3 expression was reduced in P106-HLCs when compared with PHH, they were superior to 33D6-, H9-, or Man12-derived HLCs. In comparison, hESC-HLCs barely showed any TLR3 expression (Figure S2). Secondly, using the iPSC line P106 for all further studies, we focused on a cell line that, unlike the line 33D6, does not possess genetic insertions, thereby ruling out transgene position effects on cell behavior. 33D6-derived HLCs have been shown to be less metabolically functional, further justifying the focus on the P106 line [26].

### 2.3. Stimulation of HLCs with PRR Ligands

Upon stimulation with ligands targeting virus specific PRRs, HLCs generated typical anti-viral innate immune responses, which were characterised by the release of pro-inflammatory cytokines and chemokines. Activation of the RIG-I receptor by polyI:C transfection induced both IL29 (type III IFN) and IFNB1 (type I IFN) expression (Figure 2b). IL29 mRNA levels increased dramatically between 30 min and 6 h post transfection. At the 6 h time point, mRNA levels were 45 times higher compared with unstimulated cells and levels increased further until they reached a 50-fold upregulation 24 h post transfection. IFNB1 mRNA levels poorly upregulated during the first 30 min of the experiment, followed by a stronger increase until 6 h post-transfection when upregulation was 20-fold. Following polyI:C transfection, IFN-expression induced Janus kinase/signal transducer and activator of transcription (JAK/STAT) signalling, leading to the expression of IFN-stimulated genes (ISG) such as ISG15 and Mx1. Altered kinetics of ISG-expression coincided with the expression of IFN mRNA (Figure 2b). The chemokine CXCL10 is a chemoattractant released by hepatocytes under inflammatory conditions. The release of CXCL10 can be induced by signalling through RIG-I, TLR3, interferons, and TNF-α. Transfection with polyI:C, which activated RIG-I signalling, induced a 10-fold increase in CXCL10 protein over unstimulated cells (Figure 2c). Poly I:C transfection slightly reduced cell viability to 85% after 24 h of incubation, as determined by adenosine triphosphase (ATP) assay (Figure S3).

Stimulation with polyI:C, which activates TLR3, induced IL29 and IFNB1 production; however, this was lower when compared with polyI:C transfection. Interestingly, upregulation of IL29 mRNA started shortly after the cells were stimulated, the levels increased continuously until they peaked at 6 h post stimulation and decreased afterwards. In contrast to IL29, mRNA levels for IFNB1 changed little in the course of the experiment (Figure 3b). High expression levels of IL29 and low IFNB1 expression upon RIG-I and TLR3 activation in our cell line correlates with in vivo findings where HCV-infection of chimpanzees primarily induced the expression of type III IFNs [13]. As observed with RIG-I activation, IFNs expressed upon TLR3 activation induced the expression of ISGs (ISG15, Mx1). Despite that some upregulation of CXCL10 protein was induced by polyI:C stimulation, this was not statistically significant (Figure 3c). Stimulation with polyI:C did not reduce cell viability after 24 h of stimulation (Figure S3).

Stimulation of HLCs with R848 (a TLR7 ligand) and ODN2395 (a TLR9 ligand) did not evoke any immune response, as predicted by the absence of TLR7 and 9 (data not shown).

Under inflammatory conditions, PHHs have been shown to switch from a metabolic to a defense phenotype [22,23,24]. This switch is marked by the downregulation of the normal metabolic functions such as cytochrome P450 activity and albumin-production when innate immune responses become activated. To investigate whether HLCs are also able to perform this switch, we measured the activity of the CYP-subtypes CYP1A2 and CYP3A and the production of albumin and AFP under stimulation and compared this to unstimulated cells. Both polyI:C transfection (RIG-I) and stimulation (TLR3) downregulated AFP-expression and CYP1A2 activity (Figure 2d or Figure 3d). While polyI:C stimulation had quite a dramatic effect on CYP1A2, with a three-fold decrease in its activity, CYP3A was completely unaffected. Transfection with polyI:C downregulated CYP1A2 activity 1.3-fold.

### 2.4. Upregulation of TLR3 mRNA and Signalling upon Stimulation

**Figure 4:**
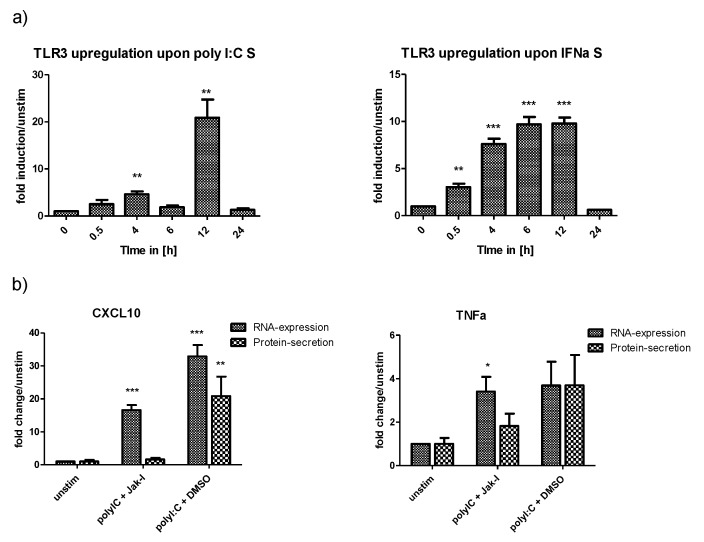
Upregulation of TLR3 expression and function under inflammatory conditions. (**a**) The viral ligand polyI:C induces upregulation of TLR3 mRNA. Medium was supplemented with polyI:C or IFN-α to mimic an inflammatory environment; TLR3 mRNA was measured by qPCR at different times after stimulation. TLR3 mRNA was significantly upregulated in a time-dependent manner when the cells were stimulated with IFN-α or polyI:C. While IFN-α treatment induced a gradual upregulation of TLR3 mRNA, which peaked at 12 h post-stimulation, polyI:C induced TLR3 mRNA abruptly raised levels at 12 h (*N* = 3). (**b**) Gene and protein expression of CXCL10 and TNF-α in polyI:C stimulated cells in the presence of Jak-I. Cells were pretreated with 10 µM Jak-I or vehicle control DMSO for 1 h. The medium was replaced with polyI:C containing medium with either Jak-I or vehicle control and the cells were cultured for a further 16 h before mRNA and protein levels of CXCL10 and TNF-α were measured by qPCR and ELISA, respectively. Despite inhibition of Janus kinase/signal transducer and activator of transcription (JAK/STAT) signalling by Jak-I, polyI:C stimulated cells showed significant induction of CXCL10 and TNF-α gene-expression, indicating that polyI:C-detection by TLR3 and associated nuclear factor kappa-light-chain-enhancer of activated B cells (NF-kB) signalling plays a role in initiating anti-viral innate immune responses (*N* = 3). It has to be noted that the potent induction of CXCL10 mRNA in Jak-I treated cells is in part owing to the IFN-independent induction of CXCL10 expression [27,28,29], indicating that TNF-α is the more reliable readout. Results are shown as mean +/− SEM. *p < 0.05, ** *p* < 0.01, and *** *p* < 0.001. TLR = toll-like receptor, S = stimulation, IFN = interferon, CXCL10 = C-X-C motif chemokine 10, TNF = tumor necrosis factor alpha, DMSO = dimethylsulfoxide.

Activation of a PRR by its ligand activates a positive or a negative feedback loop. These feedback loops have regulating effects on the immune response and can enhance pathogen-associated molecular pattern (PAMP) detection by receptor upregulation or prevent an immune overreaction by receptor downregulation. To evaluate whether HLCs possess a functional TLR3-response, we investigated upregulation of TLR3 mRNA under different stimulation conditions. Interestingly, IFN-α and polyI:C stimulation were the strongest stimuli for the upregulation of TLR3 mRNA (Figure 4a). PolyI:C induced particularly strong upregulation of TLR3 mRNA 12 h post stimulation, with a 20-fold increase in TLR3 mRNA levels over unstimulated cells. In contrast, IFN-α induced a continuous increase in TLR3 mRNA, which peaked at 12 h. Compared with polyI:C stimulation, IFN-α was less potent in inducing the expression of TLR3 mRNA, with a maximum increase of 10-fold over unstimulated cells. PolyI:C is a direct ligand for TLR3, while IFN-α is expressed downstream of TLR3-signalling. In contrast, polyI:C transfection, which activates RIG-I signalling, had an only minor effect on TLR3 mRNA expression (data not shown). 

**Figure 5:**
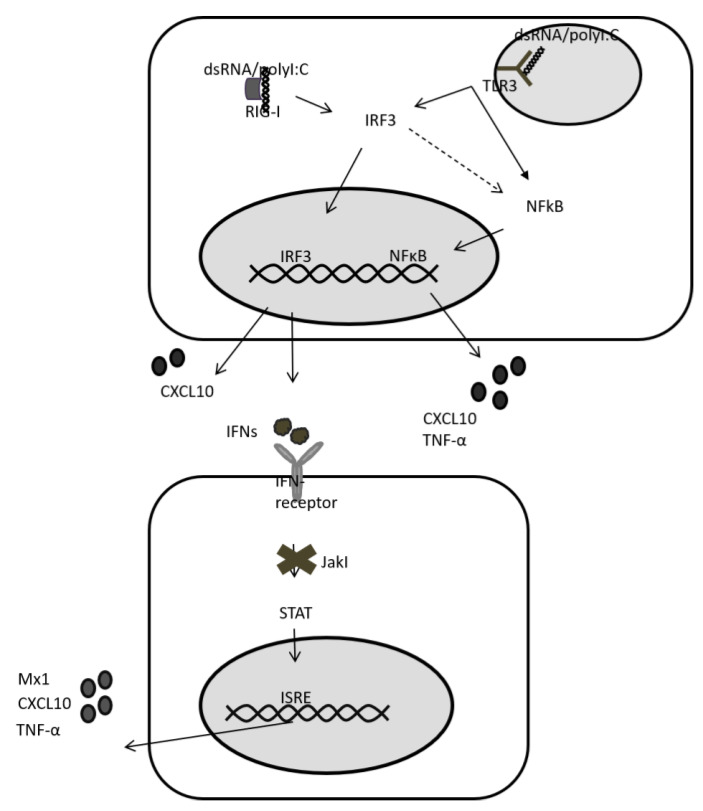
RIG-I and TLR3 signalling in d20 HLCs. Both RIG-I and TLR3 signal via interferon regulatory factor 3 (IRF3), leading to IFN release. IFNs signal via JAK/STAT to induce expression of ISGs (Mx1, CXCL10, TNF-α). When JAK/STAT signalling is blunted by a Jak-inhibitor (JakI), RIG-I signalling only induces IFN-expression. In contrast, TLR3 also signals via NFkB, which accounts for the expression of CXCL10 and TNF-α in a situation where IFN-signalling is absent. While in immune cells RIG-I also signals via NFkB, data on hepatocytes are conflicting. As such, Lee et al. have reported a lack of a significant effect of RIG-I and MDA5 signalling on TNF-a expression in Huh7 cells [30]. Note that CXCL10 can also be induced by IFN-independent signalling owing to an IRF3-response element in the CXCL10 promotor. ISRE = Interferon-stimulated response element.

To further study the presence of TLR3, we analysed gene expression upon TLR3 signalling. TLR3 signals via interferon regulatory factor 3 (IRF3) and NFkB to induce IFN-expression and expression of CXCL10 and TNF-α, respectively. (Figure 5). In contrast, RIG-I only induces the expression of IFNs. IFNs, and then signal via JAK/STAT to induce expression of ISGs including CXCL10 and TNF-α. As such, differentiating between TLR3- and RIG-I-induced gene expression can be difficult, and the abundance of RIG-I in hepatocytes easily overshadows TLR3 signalling. Therefore, we decided to block IFN-signalling and ISG expression with a JAK/STAT inhibitor (JakI) (Figure 5). To confirm successful blunting of IFN-signalling by JakI, we first stimulated JakI treated HLCs with IL29. Gene expression analysis for CXCL10 and Mx1 showed that JakI treatment of cells successfully abolished JAK/STAT signalling and ISG-expression (Figure S4). We then analysed gene- and protein-expression of CXCL10 and TNF-α in polyI:C stimulated HLCs in the presence of JakI. Interestingly, JakI did not abolish CXCL10 or TNF-α gene expression in polyI:C stimulated cells (Figure 4b). Indeed, in the presence of JakI, mRNA levels of both CXCL10 and TNF-α were still significantly upregulated compared with unstimulated cells. JakI treated cells, where RIG-I signalling is suppressed and polyI:C is only detected by TLR3, expressed lower levels of CXCL10 and TNF-α mRNA when compared with vehicle-treated cells, where RIG-I signalling is active. This observation emphasises the importance of suppressing RIG-I and JAK/STAT signalling when analysing TLR3 functionality. While both TNFα and CXCL10 mRNA were upregulated in the presence of JakI, CXCL10 mRNA levels were higher compared with TNFα mRNA levels. It cannot be excluded that this difference is caused by IFN-independent upregulation of CXCL10 mRNA [27,28]. In contrast to mRNA expression, neither CXCL10 nor TNF-α protein was significantly upregulated under Jak-inhibition (Figure 4b).

### 2.5. Interferon Stimulation of HLCs

**Figure 6:**
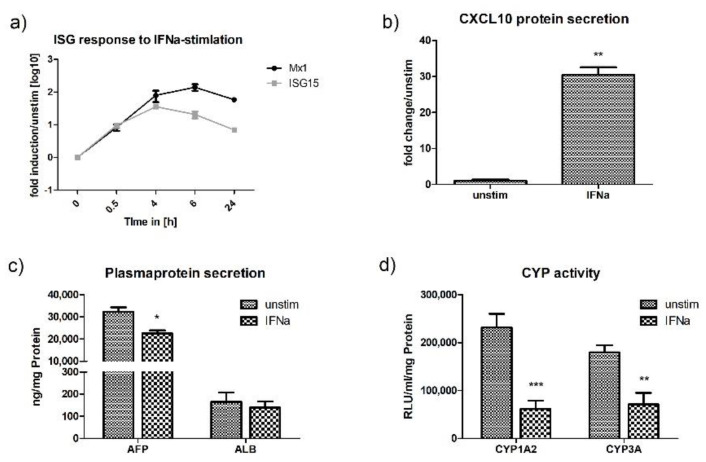
IFN-α induces anti-viral activity in P106-HLCs, which is accompanied by an immunometabolic switch. (**a**) P106-HLCs demonstrate strong innate immune activity upon stimulation with IFN-α. Stimulation with IFN-α induced ISG-expression (ISG15, Mx1) in a time-dependent manner, as determined by qPCR (*N* = 3). (**b**) Expression of CXCL10 protein upon IFN-α stimulation. Stimulation with IFN-α induced CXCL10 protein expression as determined by ELISA at the 24 h time point (*N* = 3). (**c**) Effect of innate immune activation on hepatocytes metabolic activity. Cells were stimulated with IFN-α for 16 h before CYP-activity and plasma protein production was measured and compared with unstimulated cells. The generation of an inflammatory environment as determined by upregulation of cytokines and chemokines was accompanied by the downregulation of plasma protein production (AFP, ALB) and (**d**) cytochrome P450 (CYP1A2, CYP3A) in order to redirect intracellular energy into defense strategies (*N* = 3). Results are shown as mean +/− SEM. * *p* < 0.05, ** *p* < 0.01, and *** *p* < 0.001. IFN = interferon, ISG = interferon stimulated genes, CXCL10 = C-X-C motif chemokine 10, AFP = alfa fetoprotein, ALB = albumin, CYP = cytochrome P450.

**Figure 7:**
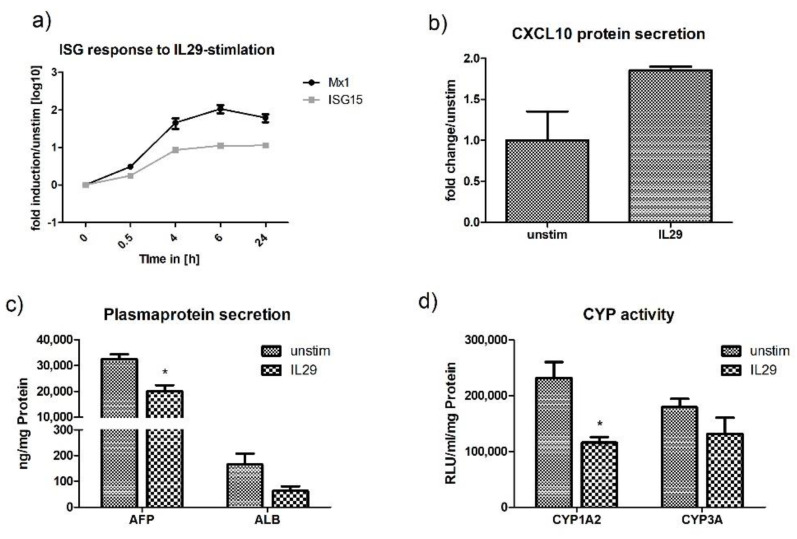
IL29 induces anti-viral activity in P106-HLCs, which is accompanied by an immunometabolic switch. (**a**) P106-HLCs mount strong innate immune responses upon stimulation with the type III IFN IL29. Stimulation with IL29 induced ISG-expression (ISG15, Mx1) in a time-dependent manner, as determined by qPCR (*N* = 3). (**b**) Expression of CXCL10 protein upon IL29 stimulation. CXCL10 protein levels in the supernatants were measured by ELISA 24 h after stimulation and confirmed the expression of CXCL10 protein (*N* = 3). (**c**) Effect of innate immune activation on hepatocytes metabolic activity. Cells were stimulated with IL29 for 16 h before CYP-activity and plasma protein production was measured and compared with unstimulated cells. The generation of an inflammatory environment as determined by upregulation of cytokines and chemokines was accompanied by the downregulation of plasma protein production (AFP, ALB) and (**d**) cytochrome P450 (CYP1A2, CYP3A) in order to redirect intracellular energy into defense strategies (*N* = 3). Results are shown as mean +/− SEM. * *p* < 0.05. IL29 = interleukin 29, ISG = interferon stimulated genes, CXCL10 = C-X-C motif chemokine 10, AFP = alfa fetoprotein, ALB = albumin, CYP = cytochrome P450.

To confirm functionality of the interferon pathway, HLCs were directly stimulated with IFN-α (type I IFN) and IL29 (type III IFN). These stimulation experiments induced ISG-expression (ISG15, Mx1, CXCL10), with IFN-α acting as a slightly stronger stimulant compared with IL29, while cell viability was not affected by neither stimulant (Figure S3). In comparison with polyI:C stimulation/transfection, when ISG-expression occurred downstream of IFN-expression and JAK/STAT-signalling, direct stimulation with IFN-α and IL29 resulted in a more immediate ISG-response (Figure 6a or Figure 7a). In accordance with the capacity of IFN-α to activate a strong innate immune response in HLCs, as defined by a strong upregulation of CXCL10 protein as well as ISG15 and Mx1 mRNA, IFN-α also downregulated CYP-activity (Figure 6b,d). The activities of CYP1A2 and CYP3A were reduced 4-fold and 2.5-fold, respectively, the most dramatic effect seen in our experiments. In addition, supplementing the media of HLCs with IFN-α also impaired the production of AFP (1.4-fold) (Figure 6c).

Similar to stimulation and transfection with polyI:C, stimulation with IL29 specifically induced the upregulation of CXCL10 and suppressed the production of AFP and the activity of CYP1A2 (Figure 7b–d). As treatment of HLCs with polyI:C (transfection and stimulation) primarily induced the production of IL29 (Figure 2a), the similar effect of IL29, polyI:C stimulation, and poly:C transfection on the metabolic activity of HLCs is not surprising and can probably be attributed to the effect of IL29 on CYP-activity and AFP-production.

### 2.6. TNF Alpha Stimulation of HLCs

**Figure 8:**
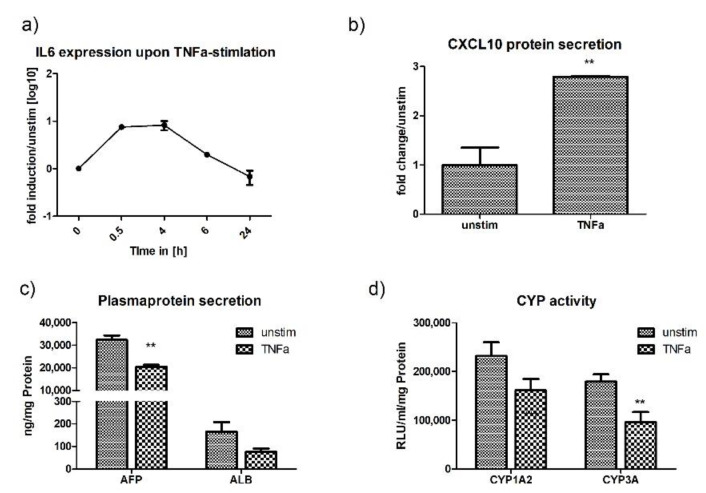
TNF-α induces anti-viral activity in P106-HLCs, which is accompanied by an immunometabolic switch. (**a**) P106-HLCs mount strong innate immune responses upon stimulation with TNF-α. Stimulation of P106-HLCs with TNF-α induced IL6-expression in a time-dependent manner, with IL6-levels peaking directly after addition of TNF-α to the medium (*N* = 3). (**b**) Expression of CXCL10 protein upon TNF-α stimulation. ELISA for CXCL10 24 h after the addition of TNF-α to the medium revealed a strong activation of CXCL10 protein expression upon TNF-α treatment (*N* = 3). (**c**) Effect of innate immune activation on hepatocytes metabolic activity. Cells were stimulated with TNF-α for 16 h before CYP-activity and plasma protein production was measured and compared with unstimulated cells. The generation of an inflammatory environment as determined by upregulation of cytokines and chemokines was accompanied by the downregulation of plasma protein production (AFP, ALB) and (**d**) cytochrome P450 (CYP1A2, CYP3A) activity, indicating a metabolic shift required to redirect intracellular energy into defense strategies (*N* = 3). Results are shown as mean +/− SEM. ** *p* < 0.01. IL6 = interleukin 6, TNF-α = tumor necrosis factor alpha, CXCL10 = C-X-C motif chemokine 10, AFP = alfa fetoprotein, ALB = albumin, CYP = cytochrome P450.

HLCs responded to TNF-α stimulation with the release of IL6 and CXLC10. The IL6 response occurred promptly, with a peak as early as 30 min post-stimulation, where mRNA levels were increased 10-fold (Figure 8a). Further, CXCL10 protein levels in the supernatant were significantly upregulated after 24 h, while cell viability was only slightly reduced to 91% (Figure 8b and Figure S3). The release of inflammatory cytokines and chemokines coincided with a significant downregulation of normal metabolic functions. When stimulated with TNF-α, we observed a significant downregulation of AFP (1.5-fold) and CYP3A (1.8-fold) in HLCs (Figure 8c,d). TNF-α was the only stimulant with a preference for CYP3A. Overall, these results show that the activation of an anti-viral innate immune response suppresses the normal metabolic activity of HLCs, and the level of metabolic downregulation is proportional to the innate immune response induced.

## 3. Discussion

In this study, we sought to evaluate the anti-viral innate immune potential of iPSC-derived HLCs, expanding on previous studies to include TLR expression and function. To this aim, we sought to mimic the innate immune response to RNA viruses. We looked for expression of the receptors for viral PAMPs, including cytoplasmic and endosomal PRRs, focusing in particular on the regulation of TLR3 expression in HLCs, an important, but underrated detector of RNA-virus infection in PHHs. We tested the ability of HLCs to respond to ligands for PRRs (RIG-I/MDA5; TLR3, 7, 9); type I IFN, the principle activator of innate anti-viral genes; type III IFN, the main IFN expressed in hepatocytes upon viral infection; and TNF-α, an inflammatory cytokine produced early in viral infection. To determine whether HLCs undergo a specific metabolic switch, a crucial step for the activation of an innate immune response in PHHs, we analysed how the induction of anti-viral innate immune responses affects the normal metabolic functions of HLCs. 

IPSC- and hESC-lines were differentiated into hepatocytes using a well-established protocol [26,31,32]. At the end of the differentiation protocol, HLCs were metabolically active as determined by cytochrome P450 activity, which was comparable to CYP-activity in male PHHs [7]. Further, HLCs co-expressed albumin, a marker of mature hepatocytes, as well as AFP, showing that the cells still retain some elements of an immature phenotype. While many protocols have focused on improving cell maturation, we believe that the immature phenotype of HLCs may be beneficial for studies of certain hepatotropic viruses such as HCV, which poorly infects mature hepatocytes [33,34,35,36].

Comparison of viral ligand receptor expression in the four stem cell lines and their differentiated counterparts showed a general trend towards receptor downregulation in the course of differentiation. Despite this trend, absolute levels of receptor expression in HLCs were different between the cell lines used and were higher in PHH. Together with the differences in basal CYP activity, this observation shows that the properties of PSC-derived HLCs differ largely between cell lines, an observation that is further supported by comparison to previous studies [11]. When we compared TLR gene expression in HLCs derived from hESCs and iPSCs, we found that only P106-derived HLCs expressed appreciable levels of TLR3 mRNA. This finding is in accordance with studies by other authors who detected no or low levels of TLR mRNAs in PSC and their derived somatic cells [37,38].

We next recreated an inflammatory environment by adding IFN-α or polyI:C to the culture medium. Interestingly, this induced a further upregulation of TLR3 mRNA in P106-derived HLCs. We propose that the induction of an antiviral state in HLCs not only consists of the release of antiviral proteins and regulation of the constitutively expressed PRRs, but also increases the repertoire of PRRs ready to detect incoming viruses. While RIG-I signalling underlies a negative feedback loop to avoid an excess immune response, the endosomal PRR TLR3 becomes upregulated [39,40,41,42]. It is particularly interesting that polyI:C stimulation, which directly targets the receptor, was particularly efficient in upregulating TLR3 mRNA, demonstrating a positive feedback loop induced by the presence of a direct TLR3-ligand [43]. We believe this could be a further mechanism of hepatocytes to shift from a more tolerogenic phenotype to defense mode, which is observed upon activation of an anti-viral immune response.

Exposure of P106-derived HLCs to different pro-inflammatory stimuli evoked innate immune responses. The observed immune responses were typical for hepatocytes and HLCs with a strong and immediate type III and a weaker and delayed type I IFN-response upon RIG-I and TLR3 activation. This pattern has been observed by others and was confirmed in this study. The use of synthetic PRR ligands rather than life virus infection ensured that all cells were equally affected by our stimulants. This allowed us to analyse the activation of each innate immune receptor in isolation. In contrast, virus infection of HLCs only results in the successful infection of small foci of cells [9]. The infected cells then activate an innate immune response, which induces an antiviral state in neighbouring cells. 

Stimulation versus transfection of polyI:C results in different locations of the stimulant (cytoplasm vs. endosome), thus allowing its detection by different PRRs (RIG-I for cytoplasmic detection, TLR3 for endosomal detection). However, stimulation with polyI:C for endosomal detection inevitably leads to some leakage into the cytoplasm followed by detection by RIG-I. In addition, both TLR3-signalling and downstream RIG-I signalling result in the expression of the same set of genes (Figure 5). Hence, TLR3 signalling in hepatocytes is often masked by the strong and immediate RIG-I response. A functional TLR3 pathway was observed with upregulation of the receptor following IFN-α and polyI:C stimulation. To further study the possibility of a functional TLR3 pathway in HLCs, we blocked downstream signalling of RIG-I by JakI in order to avoid the effect of polyI:C leakage into the cytoplasm. TNF-α gene expression remained high when the IFN pathway is blunted, indicating the TLR3 response is functional.

The differences in the ability to activate RIG-I and TLR3 might be explained by the different subcellular locations of the two receptors. RIG-I is highly expressed in the cytoplasm of healthy hepatocytes. In contrast, TLR3, upon upregulation, is located in the endosome, where it is important at later times during infection [44,45,46]. Upon virus endocytosis, virus particles translocate to the cytoplasm where uncoating and viral replication occur. During this stage, viral PAMPs are detected by cytoplasmic PRRs and an innate immune response is initiated, which also induces upregulation of specific TLRs such as TLR3. During replication, some viral replication products are endocytosed via the cell’s recycling mechanism, where they are now presented to endosomal TLR3. Considering the tightly regulated mechanism of TLR3-expression in hepatocytes, we believe that the detection of a single stimulant by TLR3 in our model is not sufficient to induce a robust innate immune response. While induction of cytokine-expression on the gene level confirms the upregulation of TLR3 in HLCs, the rather immunotolerant character of the hepatocyte prevents efficient translation into protein under these conditions. Moreover, while inhibiting RIG-I-induced IFN-signalling allowed us to exclude false-positive results when analysing TLR3-signalling, it is possible that the lack of a strong initial RIG-I-induced immune response prevented efficient TLR3-upregulation necessary for a robust TLR3-induced immune response. It will be interesting to compare TLR3 upregulation and immune response induction in HLCs generated from virus-susceptible and -resistant people in the future. On the basis of the higher rate of autoimmune diseases in HCV-resistant individuals, it will also be interesting to see whether TLR-responses are more easily activated in these individuals.

Stimulation of P106-HLCs with TNF-α induced the expression of the pro-inflammatory cytokines IL6 and CXCL10. TNF-α, a major mediator of systemic inflammation, also has a key role in liver regeneration [47,48] and plays important roles in RNA virus infection, ranging from stimulating hepatocytes to express cytokines to the disruption of tight junctions and promotion of HCV entry [49,50]. Responsiveness of HLCs to TNF-α, therefore, opens up various opportunities to study the diverging roles of TNF-α during virus–hepatocyte interactions.

A major feature of immune responses in primary hepatocytes is their switch from a metabolic to an immune active state under inflammatory conditions. During homeostasis, metabolism is the main function of healthy hepatocytes, which are responsible for vital processes such as glycogen synthesis, serum protein production, and detoxification. However, under inflammatory conditions, normal metabolic activities such as albumin production and cytochrome P450 activity are downregulated, while innate immune responses are upregulated. It has been suggested that the downregulation of CYP-activity in inflammation is a mechanism to prevent further cellular damage, for example, through the production of reactive oxygen species (ROS) [51]. A role of CYP-downregulation in the acute phase has also been suggested as heme is used by CYP-proteins for drug-detoxification and by the acute phase in inflammation [51]. In this study, we found that HLCs highly resemble PHHs with regard to CYP downregulation. We observed effects of different stimuli on CYP1A2 and CYP3A activity. In our study, TNF-α specifically downregulated CYP3A-activity, while CYP1A2-activity was not significantly altered. The specific effect of TNF-α on CYP3A-activity is also known in PHHs and has been attributed to the induction of the acute phase by TNF-α [22,52]. In addition to CYP downregulation, HLCs also showed reduced albumin and AFP production when innate immune pathways became activated. Overall, we observed that the level of albumin and AFP downregulation correlated with the strength of the innate immune response induced by a specific stimulant. These observations show that iPSC-HLCs are able to adapt to the requirements of an inflammatory environment by downregulating their metabolic activity and redirecting intracellular energy into defense strategies. The ability of HLCs to undergo a metabolic switch, similar to that of PHHs, is an important factor for studies on inter-individual differences in susceptibility to disease. The efficiency of an anti-viral innate immune response is based on the presence and functionality of PRRs, as well as the ability to redirect cellular energy into defense mechanisms. In this study, we could show that HLCs are able to perform such metabolic switches and represent an important tool for studies on host-pathogen interactions and inter-individual differences in susceptibility to disease.

## 4. Materials and Methods

### 4.1. Reagents

mTeSr medium was purchased from Stem Cell Technologies; RPMI, 50x B27 supplement, knockout DMEM, knockout serum replacement, glutaMAX, β-mercaptoethanol, penicillin/streptomycin, and hepaotZYME Serum-free medium (SFM) were purchased from Life Technologies. Recombinant mouse Wnt3a was from R&D, while activin A, hepatocyte growth factor (HGF), oncostatin M (OSM), TNF-α, interleukin 29 (IL29), and IFN-α were from PeproTech. PolyI:C, R848, and ODN2395 were from InvivoGen. PHH RNA from three different donors was from 3H Biomedical.

### 4.2. Hepatocyte Differentiation of iPSCs

The iPS-lines P106 (JHU106i, WiCell) and 33D6 [26] and the hES-lines H9 (WA09, WiCell) and Man12 (University of Manchester) were cultured on Laminin521-coated wells (Biolamina) under feeder-free conditions [6,7]. Cells were cultured in mTeSr medium until they reached 80% confluence. One day prior to differentiation, 50,000 cells/cm2 were seeded on coated 96-well culture plates in the presence of 10 µM Rock Inhibitor. Pluripotent stem cells were differentiated in large scale using an established protocol [7,26,31,32]. Briefly, endoderm formation was induced by RPMI/1x B27 supplemented with 1 µl/mL Activin A and 5 µl/mL Wnt3A for three days. Subsequently, cells were cultured in the presence of 1% dimethyl sulfoxide (DMSO) for five days to achieve hepatoblast formation. For hepatic maturation, cells were cultured in HepatoZYME in the presence of 1 µL/mL HGF and OSM until d20.

### 4.3. Immunofluorescence Staining

For immunofluorescence staining of the hepatocyte markers, albumin, AFP, and hepatocyte nuclear factor 4 alpha (HNF4α), cells were fixed in 4% paraformaldehyde (PFA) for 15 min at room temperature. The fixed cells were washed twice with phosphate buffered saline + 0.1% tween (PBST) and blocked with 10% bovine serum albumin (BSA) in PBST for 1 h at room temperature. Cells were washed 3x with PBST and the primary antibody was added (PBST + 1% BSA) and incubated at 4 °C overnight. Primary antibodies used are listed in Table S2. The primary antibody was removed and the cells were washed three times with PBST before incubating with the appropriate secondary antibody (PBST + 1% BSA) for one hour at room temperature. Before visualisation, cells were counterstained with Hoechst 33,342 (NucBlueTM Live Ready ProbesTM Reagent). Images were acquired on an Olympus IX81 fluorescence microscope.

### 4.4. RT-qPCR

Total RNA was isolated using Trizol (LifeTechnologies) and 1 µg of RNA was transcribed into cDNA (Omniscript RT kit, Qiagen). qPCR was performed using Taqman probes (ThermoFisher) on a Roche Light Cycler 460 II. Primers used are listed in Table S1. Gene expression levels were normalised to ß2-microglobulin and compared to PHH RNA mixed from three donors

### 4.5. Stimulation

To investigate specific innate immune pathways important in RNA virus detection and response, d20 HLCs were stimulated with low molecular weight (LMW) polyI:C at 100 µg/mL (TLR3), IL29 at 100 ng/mL and IFN-α at 100 ng/mL, TNF-α at 50 ng/mL R848 (TLR7) at 100 ng/mL, or ODN 2395 at 5µM (TLR9). Cytoplasmic presence of polyI:C for detection by RIG-I was achieved by chemical transfection at 10 ug/mL (Lipofectamin 3000, LifeTechnologies). Cells were collected in Trizol after 0, 4, 6, 12, and 24 h of incubation.

### 4.6. Enzyme-Linked Immunosorbent Assay (ELISA)

ELISAs for CXCL10 and TNF-α were performed using the human IP-10 TMB ELISA and human TNF-α TMB ELISA kits (Peprotech), respectively, according to the manufacturer’s instructions. Human AFP and human albumin ELISA were from Alpha Diagnostics and ELISAs were performed according to the manufacturer’s instructions. All protein expression levels were normalised to miligram of protein as determined by bicinchoninic acid assay (BCA) assay (ThermoFisher).

### 4.7. Janus Kinase/Signal Transducer and Activator of Transcription (JAK/STAT)-Pathway Inhibition

To determine whether increased TLR3-expression was mediated by the JAK/STAT pathway, cells were preincubated with a Jak-inhibitor (10µM, Merck Millipore). After one hour, the medium was changed into polyI:C containing medium supplemented with 10 µM Jak-I and cells were cultured for 24 h before cells and supernatants were collected for further analysis.

### 4.8. Cytochrome P450 Assays

Cytochrome P450 activity was measured using the Promega P450-Glo CYP3A4 and Promega P450 CYP1A2 Assay kits according to the manufacturer’s instructions. Data were normalised to milligram of protein per milliliter.

### 4.9. Statistical Analysis

GraphPad Prism 5.0 software was used for statistical analysis. All data are given as mean and standard error of the mean. Differences between independent groups were analysed by unpaired Student’s t-test. Statistical significance was * for *p* < 0.05, ** for *p* < 0.01, and *** for *p* < 0.001.

## 5. Conclusions

In this study, we found that iPSC-derived HLCs express not only cytoplasmic PRRs, but also TLR3. TLR3 mRNA expression was low but could be upregulated by the recreation of an inflammatory environment. HLCs are able to respond to viral ligands by mounting an anti-viral innate immune response. However, TLR3 signalling alone was insufficient to induce a robust innate immune response. This shows the importance of the interplay of several PRRs to detect viral infection at different levels to induce a strong and efficient anti-viral innate immune response. Activation of innate immune activity in HLCs was accompanied by the downregulation of normal metabolic functions such as cytochrome P450 activity and albumin and AFP secretion. This shows that HLCs are able to efficiently adapt to an inflammatory environment and have intact innate anti-viral potential.

## Supplementary Materials

Supplementary materials can be found at https://www.mdpi.com/1422-0067/21/11/3831/s1.

## Abbreviations

AFP	Alpha-fetoprotein
ALB	Albumin
CXCL10	CXC-motif chemokine 10
CYP	Cytochrome
DMSO	Dimethylsulfoxide
ELISA	Enzyme-linked immunosorbent assay
HBV	Hepatitis B virus
HCV	Hepatitis C virus
hESC	Human embryonic stem cell
HLC	Hepatocyte like cell
HNF4a	Hepatocyte nuclear factor 4 alpha
IFN	Interferon
IL	Interleukin
IRF3	Interferon regulatory factor 3
JAK/STAT	Janus kinase/signal transducer and activator of transcription
MDA5	Melanoma differentiation antigen 5
NFkB	Nuclear factor ‘kappa-light-chain-enhancer’ of activated B-cells
PAMP	Pathogen associated molecular pattern
PSC	Pluripotent stem cell
RIG-I	Retinoic acid inducible gene I
PRR	Pattern recognition receptor
TLR	Toll-like receptor
TNFa	Tumor necrosis factor alpha
