# Supplementary material for: Human PSC-Derived Hepatocytes Express Low Levels of Viral Pathogen Recognition Receptors, but Are Capable of Mounting an Effective Innate Immune Response
Source: Int J Mol Sci. 2020 May 28;21(11):3831. doi: 10.3390/ijms21113831 (PMC7312201; doi:10.3390/ijms21113831)
Supplement: Supplementary file 1 [file ijms-21-03831-s001.pdf]

Supplementary Material

Table 1. Primers for qPCR.

Gene	Primer
ß2-microglobulin	Hs00984230_g1
RIG-I (DDX58)	Hs01061436_m1
Mda5 (IFIH1)	Hs00223420_m1
TLR3	Hs01551079_g1
TLR7	Hs00152971_m1
TLR9	Hs00152973_m1
IFNL1	Hs00601677_g1
IFNB1	Hs01077958_s1
CXCL10	Hs00171042_m1
IL6	Hs00174131_m1
ISG15	Hs00192713_m1
Mx1	Hs00895608_m1
TNF-α	Hs00174128_m1

Table 2. Antibodies for immunofluorescence.

Antibody	Company	Raised in	Cat#
Albumin	Sigma	Mouse	A6684
AFP	Abcam	Mouse	Ab75705
HNF4a	Santa Cruz	Rabbit	

a)

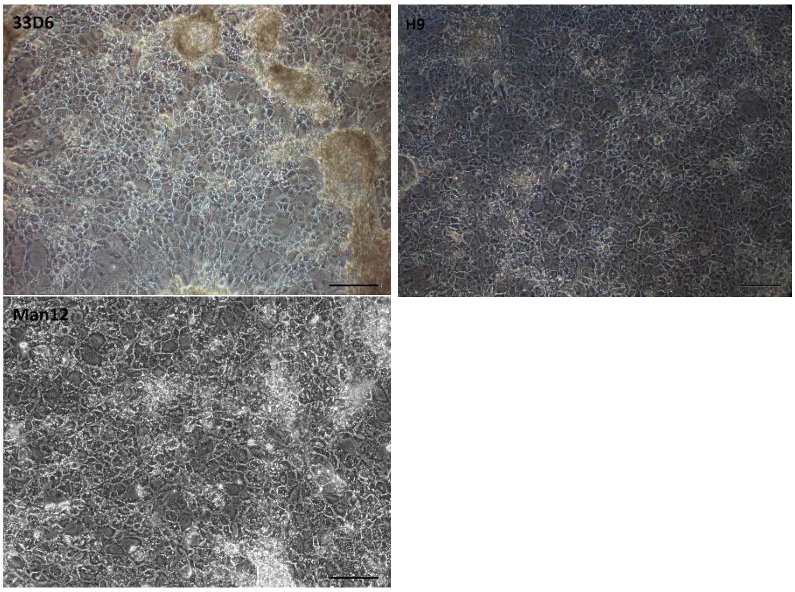

b)

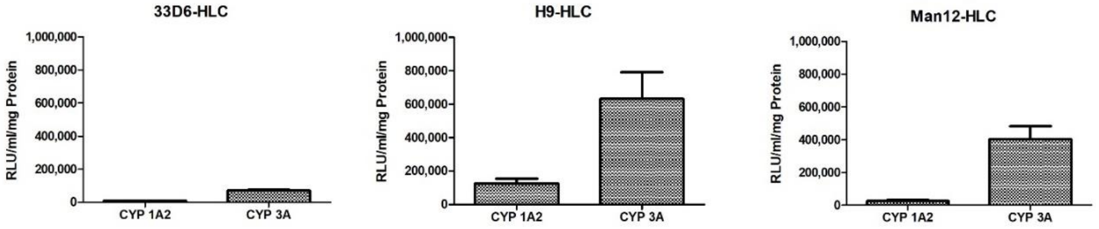

Figure 1. Characterisation of 33D6-, H9- and Man12-derived HLCs. a) Representative light phase microscopy picture of HLC morphology (10x magnification). At the end of the differentiation protocol, HLCs had acquired a cobblestone morphology with a large cytoplasm to nucleus ratio. Scale bars, 50μm. b) Metabolic activity. The metabolic activity of HLCs was determined by measuring the

activity of CYP1A2 and CYP3A. CYP-activity was measured in RLU and normalized to ml and mg of total protein (n=3 for 33D6, n=8 for H9 and Man12). 33D6-derived HLCs show a reduced metabolic function compared to P106-HLCs (Fig. 1b) and hESC-HLCs. CYP1A2 activity in particular was low compared to the other cell lines profiled. CYP-activity of H9-derived HLCs were comparable to PHH CYP-activity published by Cameron et al., (7). Results are shown as mean +/- SD.

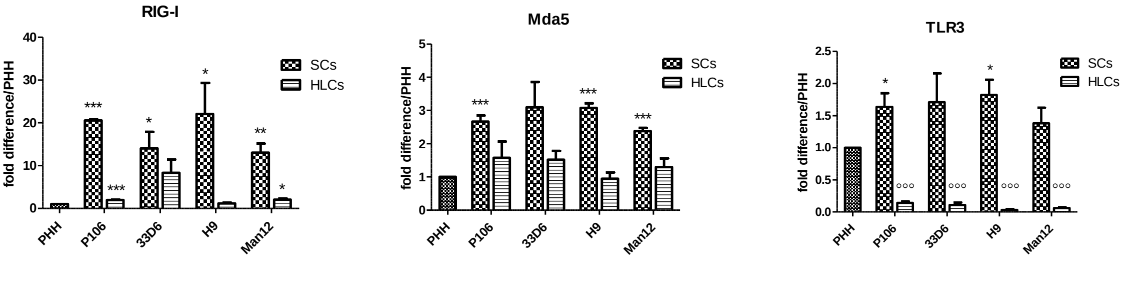

Figure 2. Comparison of PRR gene expression between different stem cell lines in the undifferentiated state and after differentiation into HLCs. After differentiation P106-, H9- and Man12-derived HLCs express similar levels of RIG-I. Mda5 expression is only slightly upregulated compared to PHH. Most importantly, despite the significant lower expression of TLR3 in HLCs derived from all cell lines, expression is significantly higher in P106-derived HLCs than in H9- or Man12-derived HLCs, which makes this cell line more suitable for studies on anti-viral innate immunity. Results are shown as mean +/- SD (n=3) * represents significantly higher expression compared to PHH, ° represents significantly lower expression compared to PHH.

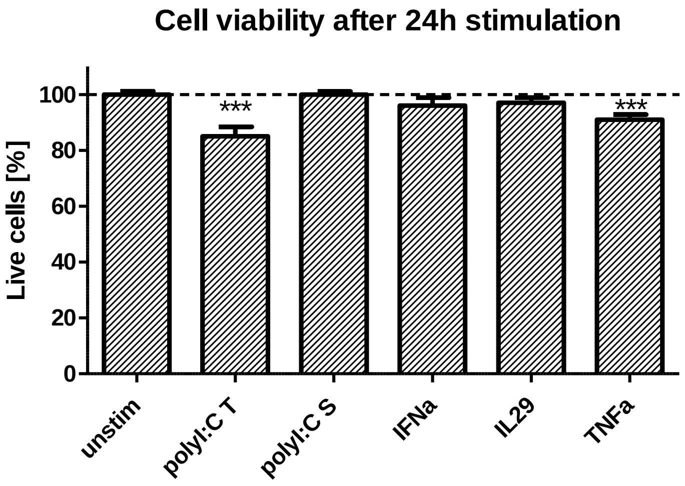

Figure 3. Cell viability after 24h of stimulation/transfection. Cells were transfected with polyI:C or stimulated with polyI:C, IFN-α, IL29 or TNF-α and cell viability was measured after 24h by ATP assay. Transfection with polyI:C and stimulation with TNF-α slightly reduced cell viability to 85% and 91%, respectively, while polyI:C stimulation, IFN-α and IL29 did not affect cell viability (n=8). Results are shown as mean +/- SD. ATP = adenosine triphosphate.

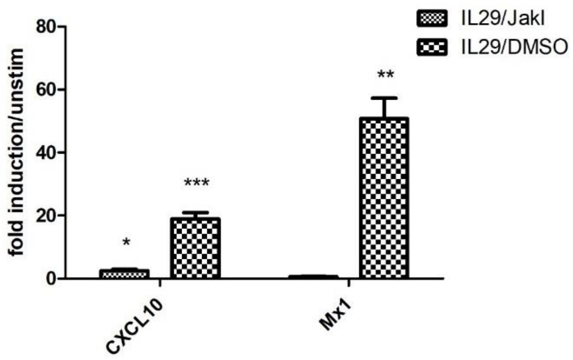

Figure 4. Gene-expression in IL29-stimulated HLCs in the presence of Jak-I and DMSO control to verify Jak-I specificity. Cells were either preincubated with 10μM Jak-I or DMSO for 1h before stimulation with IL29 in the presence of Jak-I or DMSO, respectively. 16h post-stimulation RNA was isolated; CXCL10 and Mx1 expression was evaluated by qPCR to confirm successful JAK/STAT inhibition by Jak-I (n=3). Results are shown as mean +/- SD.
